# Developmental genetics with model organisms

**DOI:** 10.1073/pnas.2122148119

**Published:** 2022-07-18

**Authors:** Uwe Irion, Christiane Nüsslein-Volhard

**Affiliations:** ^a^Research Group: Colour Pattern Formation, Max-Planck-Institute for Biology, 72076 Tübingen, Germany

**Keywords:** *Drosophila melanogaster*, *Caenorhabditis elegans*, *Danio rerio*, *Mus muculus*, *Arabidopsis thaliana*

## Abstract

In Darwin’s and Mendel’s times, researchers investigated a wealth of organisms, chosen to solve particular problems for which they seemed especially well suited. Later, a focus on a few organisms, which are accessible to systematic genetic investigations, resulted in larger repertoires of methods and applications in these few species. Genetic animal model organisms with large research communities are the nematode *Caenorhabditis elegans*, the fly *Drosophila melanogaster*, the zebrafish *Danio rerio,* and the mouse *Mus musculus.* Due to their specific strengths, these model organisms have their strongest impacts in rather different areas of biology. *C. elegans* is unbeatable in the analysis of cell-to-cell contacts by saturation mutagenesis, as worms can be grown very fast in very high numbers. In *Drosophila*, a rich pattern is generated in the embryo as well as in adults that is used to unravel the underlying mechanisms of morphogenesis. The transparent larvae of zebrafish are uniquely suited to study organ development in a vertebrate, and the superb versatility of reverse genetics in the mouse made it the model organism to study human physiology and diseases. The combination of these models allows the in-depth genetic analysis of many fundamental biological processes using a plethora of different methods, finally providing many specific approaches to combat human diseases. The plant model *Arabidopsis thaliana* provides an understanding of many aspects of plant biology that might ultimately be useful for breeding crops.

Mendel’s discovery was essentially a result of his careful selection of just one species for his study. Scientists before him, most prominently, Darwin, collected breeding data from a large variety of species, often without clearly defined background, and often without discrimination between crosses within or between species. Therefore, the results were not comparable and did not yield more insight; on the contrary, they might have obscured valuable conclusions (1). Mendel (2), in contrast, in his 1866 paper “*Versuche über Pflanzenhybriden*” (“Experiments on plant hybrids”), begins by describing the selection of the experimental plants for his studies and why this must be “done with the greatest care if one does not wish to put the results in question from the beginning.” Mendel chose pea plants (*Pisum*) for the experiments described in his paper, for a number of good reasons; first, he made sure that a whole panel of clearly distinguishable traits bred true in the different varieties (which were available from seed shops), and he also demanded that the hybrids he wanted to study were fully viable and fertile, to allow a quantitative evaluation of the crosses. He even protected some less robust plants from being overgrown. In addition, peas are easy to cultivate and, although described as somewhat cumbersome (“*etwas umständlich*”), cross-pollination is almost always successful. Besides this primary focus on one species, he was aware that his results might be of more general validity and that peas could be a model for other plants. He stated that his conclusions based on *Pisum* needed to be confirmed, but he was convinced of the principal validity of his conclusions “since the unity of the developmental plan of organic life is beyond question” (“*da die Einheit im Entwicklungsplane des organischen Lebens ausser Frage steht*”), referring to the most important result, the reduction of the copy number of factors (“*Merkmale*”) to one in the gametes and the combination to two in the plant, and the equal contribution of maternal and paternal gametes.

Since the beginning of the 20th century, when Mendel’s work was rediscovered and brought to scientists’ attention, many different biological questions were addressed, investigating all kinds of diverse organisms in many laboratories around the world. However, only few rose to outstanding prominence as so-called “model organisms” with especially large bodies of work that accumulated over the decades and impacted large parts of biology. We will confine ourselves here to the discussion of multicellular organisms that are suitable for genetic research, fully aware that this excludes *Escherichia coli* and its bacteriophages, that laid much of the foundations of modern molecular biology. Also, fungi such as yeast and *Neurospora*, in which important aspects of metabolism and cell cycle regulation have been investigated genetically, are not discussed here due to space limitations. In addition, animals and plants that are studied mainly experimentally but that do not allow large-scale genetic approaches—*Xenopus*, chicken, rats, guinea pigs, rabbits, and, among plants, corn, tobacco, tomato, snap dragon, and rice—are not included. Essential features of the models we want to discuss here are the ease with which mutants can be obtained and propagated, the number of progeny per cross, generation time, and space requirements. Genetics allows the systematic analysis of many features in one organism in great depth, and many processes studied in detail may serve as models for other organisms, due to the homology of genes. Only a few of the many objects studied at the time of Mendel and Darwin, such as the mouse and *Drosophila*, fulfilled the criteria, and the zebrafish, the nematode *Caenorhabditis elegans*, and the plant *Arabidopsis* were later chosen as models, resulting in the autocatalytic building of a growing community of researchers who accumulated data which, in turn, attracted other researchers. Additional advantages became apparent, such as an invariant cell lineage in *C. elegans,* or small chromosome number and giant polytene chromosomes in *Drosophila* larvae, embryonic stem (ES) cells in the mouse, and small genomes with low levels of redundancy for most of the models. This led to the invention and perfection of novel techniques, for example, gene targeting, gene silencing, reverse genetics, and transgenesis, which then produced a self-sustaining cycle of further advances. Much of our understanding of biology as it developed during the 20th century is based on insights gained from these model organisms. Only with the advent of modern techniques at the beginning of the 21st century, like whole genome sequencing and genome editing using the CRISPR-Cas system, is it now possible to easily branch out from these few very successful models and carry out genetic research on a much broader evolutionary scale.

## Drosophila melanogaster.

Thomas H. Morgan chose the fruit fly, *Drosophila melanogaster*, at the beginning of the 20th century as an experimental organism, to study heredity (genetics) in the laboratory. He started with flies because of the ease with which they could be bred in the laboratory, using only little space for some half-pint milk bottles with bananas as food, and their speed of reproduction, producing a new generation every 10 to 14 d. After the emergence of the first mutants in his fly stocks, most prominently a male fly with white eyes instead of the normal dark red (3), it became apparent that the rich external morphology of *Drosophila* provided a formidable basis for many more mutants to be discovered and the study of the underlying principles of heredity. Soon the four chromosomes—two large autosomes, the X chromosome, and a tiny fourth chromosome—were identified as linkage groups. The chromosomal theory of inheritance, which had been proposed a few years earlier by Boveri (4) and Sutton (5), was experimentally verified, linking Mendel’s factors (“*Merkmale*”) to chromosomes, and sex-linked inheritance was demonstrated (3). Linear genetic maps were constructed from recombination frequencies between linked mutations (6), demonstrating that genes, a term coined in 1909 by Johannsen (7), equivalent to Mendel’s factors, were distinct and separable entities located on specific positions on chromosomes. With the help of many *Drosophila* mutants, much of the basis of classical genetics was established. Allelic series were discovered, for example, partial and complete loss-of-function alleles in the case of the *white* gene, leading to pink (*eosin*) or white (*white*) eyes, respectively, thus, showing that genes could exist in more states than just two. Chromosomal deficiencies, and thereby the exact locations of genes on chromosomes, could be visualized using the polytene chromosomes from the salivary glands of third instar larvae (8), giving a physical basis to genetic maps. The discovery that mutations could be induced by radiation or chemicals (9, 10) meant that the process could be accelerated enormously. Many stocks were constructed with chromosomes that carried not only mutations with visible phenotypes (markers) but also multiple inversions, leading to a suppression of recombination events, which made them useful as “balancer” chromosomes to keep lethal and sterile mutations in heterozygous stocks.

After the realization that DNA is the genetic material carrying the genes, the era of molecular biology started in the middle of the last century. Much research initially focused on bacteria (*E. coli*) and bacteriophages, which proved simple enough for understanding many molecular details of the genes and how they are regulated. Subsequently, the question of how genes can control the development of a complex, multicellular organism came more into focus for research, and *Drosophila* was a natural target. Molecular biologists also became interested in behavior. Seymour Benzer (11) devised simple screens separating behavioral mutants that had defects in phototaxis, circadian rhythm, or memory and learning. Thus, *Drosophila* genetics provided an excellent starting point to investigate rather complex questions of animal behavior.

With its superb genetic tools and a large collection of mutants, *Drosophila* also attracted attention from developmental biologists. Especially, so-called homeotic mutants seemed interesting, with their peculiar phenotypes characterized by very specific transformations of body parts: halteres to wings in *bithorax* mutants and antennae to leg-like outgrowths in *Antennapedia* (12). When gene technology developed in the early 1980s, *Drosophila* researchers focused on cloning the homeotic gene complexes, which led to the discovery of the homeobox, a conserved DNA-binding domain of transcription factors present in invertebrates and vertebrates (13). This created great excitement because it was the first suggestion of common developmental pathways present in very divergent groups of animals.

At the time, *Drosophila* genetics was mainly the genetics of the adult fly, which develops through three larval stages followed by pupariation and metamorphosis. The adult structures in the fly develop from imaginal discs, two-dimensional sheets of epithelial cells set aside in the early embryo, which proliferate during larval growth, while most other larval cells simply increase in volume without cell divisions. During metamorphosis, the imaginal discs, which mostly come in pairs, for example, for legs or wings, are everted, and the adult body is formed. The analysis of embryonic phenotypes was hampered by the opaqueness of the eggs. Nevertheless, some of them were described, for example, for *Notch* mutants, and, a fate map of the embryo, deduced from sectioned material, was established (14). Despite the small size of *Drosophila* eggs, transplantation of posterior pole plasm was shown to induce germ cell formation at the anterior, which suggested that “cytoplasmic determinants” for germ cell formation were present in the pole plasm (15) and other cytoplasmic determinants for the imaginal structures, which would be located in the egg cortex. However, screens for such determinants were unsuccessful.

A shift away from adult flies to the analysis of larval phenotypes together with unbiased genetic screens for mutants with larval patterning defects lead to a big boost for *Drosophila* developmental genetics. The larval cuticle, with segmentally repeated patterns of denticles and hairs, provides an excellent readout for the patterning along the anterior–posterior and dorsoventral axes. Both maternally and zygotically expressed genes are required for axis determination and patterning; mutations in both classes cause embryonic lethality and lead to distinctive phenotypes visible in the larval cuticle. Large-scale unbiased mutagenesis screens were carried out in the late 1970s and early 1980s with the aim of identifying zygotically expressed genes by analyzing the pattern defects in the cuticles produced by mutant embryos. Separate screens for the two autosomes and the X chromosome were performed (16–19) to allow the application of genetic selection systems avoiding sorting of flies in the inbreeding generations. A total of 600 mutants defining 120 genes (complementation groups) were isolated. The small number of genes indicated that most essential genes are dispensable for patterning of the larva or do not require precise temporal and spatial control of expression. The genes could be classified into groups with similar phenotypes, affecting either patterning along the anterior–posterior or the dorsoventral axis. A third gene group was required for epidermal integrity (reviewed in ref. 20).

Subsequently, similar large-scale screens were performed for maternal mutants affecting patterning of the larva: a screen in Tübingen for mutants on the third chromosome, and a screen in Princeton for mutants on the second chromosome (21–24). Maternal mutants on the X chromosome had been isolated earlier in several laboratories (25). Mutants in about 30 genes were isolated; surprisingly, they made up just four groups of genes with common phenotypes, suggesting that the genes within each group form a system that specifies those regions of the body that are absent in the corresponding mutants. One group determines the dorsoventral axis, whereas three affect the patterning along the anterior–posterior axis, where individual regions are determined largely independently. The anterior system (prototype *bicoid*) is responsible for the segmented region of head and thorax, the posterior system (prototype *oskar*) for the segmented abdomen, and a third system, the terminal system (prototype *torso*), determines the nonsegmented acron and telson. Although, biochemically, the four systems of axis determination are very different, they have some features in common: Each depends on a localized signal, which functions like a cytoplasmic determinant, and, in each, a gradient of a transcription factor is established that controls the expression of zygotic target genes in a concentration-dependent manner (reviewed in ref. 26).

During early embryonic development in *Drosophila*, nuclear divisions are not followed by cytokinesis, leading to a syncytial stage with many nuclei that are not separated by cell membranes. This allows the establishment of the anterior–posterior axis via a hierarchy of transcriptions factors encoded by zygotic genes. These, in turn, depend on maternal factors, among them the anterior determinant Bicoid, which is the first clear case of a morphogen that determines cell fates in a concentration-dependent manner. Bicoid is translated in the early embryo from anteriorly localized maternal messenger RNA (mRNA) and forms an exponential protein gradient, which regulates the expression of different target genes along the anterior–posterior axis (27). Another maternally encoded transcription factor, Dorsal, determines the expression of its target genes by a gradient of nuclear localization, which depends on extracellular signals specifically present on the ventral side of the egg (28).

Cloning and sequencing of many of the patterning genes identified in these screens was facilitated by the discovery of the transposable P element and its use for insertional mutagenesis and genetic transformation (29). The expression patterns, analyzed by in situ hybridization, revealed that early patterning of the embryo depends on the delineation of discrete zones, defined by the combination of transcription factor expression along the anterior–posterior and dorsoventral axes. The collection of zygotically expressed genes included many components of the major intercellular signaling pathways: Notch, BMP (*decapentaplegic*), EGF (*spitz*), Toll, Hedgehog, and Wnt (*wingless*). All these signaling systems are conserved and play key roles also in vertebrates. Decapentaplegic, a homolog of the TGFbeta family member BMP (30), induces several cell fates along the dorsal region of the embryo in a concentration-dependent manner; it also controls patterning in imaginal discs and is the first described secreted morphogen which distributes in the extracellular space (31). Proteins of the BMP family are components of the Spemann organizer, which is required for pattern formation in vertebrate embryos (32). In contrast, segmentation in vertebrates, which is reflected in the metameric pattern of somites along the anterior–posterior axis, occurs differently compared to *Drosophila*. Whereas anatomy or gene function often suggests where to look for homologies in developmental processes, there have also been some surprises. The Toll-dorsal pathway not only controls dorsoventral patterning in the *Drosophila* embryo but also has a prominent function in innate immunity in most metazoans, including *Drosophila* (33).

More-sophisticated screens were developed, including enhancer or suppressor screens, and overexpression screens (34). Although embryonic lethality prevents the straightforward analysis of gene function during later stages, this is possible by inducing clones of homozygous mutant cells in otherwise heterozygous animals via mitotic recombination. The adaptation of the Flp/FRT system from yeast for *Drosophila* increased the efficiency of the induction of clones. Fly strains that express the site-specific recombinase (Flp) under specific promoters (35) and other strains that carry the Flp recombination target sites (FRT) at positions close to the centromeres (36) allow assessment of the functions of early lethal genes during later processes, such as oogenesis or imaginal disk development. Specific expression of Flp was facilitated by combining it with the Gal4/UAS system of targeted gene expression, which is based on the yeast transcription factor Gal4 and its DNA binding sites (upstream activating sequences, UAS) (37), a system that is also used in other organisms such as zebrafish. The efficiency and versatility of these systems in generating homozygous mutant cell clones made *Drosophila* a multicellular organism where screens can be carried out at any stage of development in almost any cell type.

In 2000, the complete genome sequence of *D. melanogaster* was published (38), the second multicellular animal after *C. elegans* (see below). This was a tremendous achievement. Gene knockdowns with RNA interference (RNAi) became possible, and genome-wide approaches followed. Approximately 61% of disease-causing genes in humans have functional homologs in *Drosophila* (39), and the fly can be used to study the function of these genes and the consequences of mutations.

Whereas other insects besides *Drosophila* were also studied in the laboratory and even had distinctive advantages, like larger body size or eggs that are easier to manipulate [e.g., leafhoppers, *Euscelis plebejus*, in which gradients were first described (40)], none of them could rival the fly with its ever-growing body of knowledge and very elaborate genetics. One system that gained some momentum as complementary to *Drosophila* was the red flour beetle, *Tribolium castaneum*. Especially after it became clear that the mode of development in *Drosophila*, and some of the genes regulating it (e.g., *bicoid*), might be very special, comparisons to more distantly related species seemed necessary (41). The fact that RNAi could be employed to study the loss of gene function in *Tribolium* proved advantageous (42); however, the very extensive collection of *Drosophila* mutants remained unrivaled.

On a somewhat less broad evolutionary scale, the genomes of 12 *Drosophila* species became available for comparison in 2006 (43), and the number has reached 101 since. This allows many evolutionary questions to be addressed, frequently still with *D. melanogaster* as a reference point. (For a recent review on *Drosophila* developmental genetics, see ref. 44.)

## C. elegans.

In contrast to *Drosophila*, the story of *C. elegans* is one of much more planning and design. That is not to say that “luck” and unforeseen advantages did not also play a considerable part in the success of the worm. Nematodes, or roundworms, are a large group of animals, often parasites, that are present in almost all habitats on Earth. They were an important object for studies on cell lineages and chromosome segregation already at the end of the 19th century (45). However, it took several decades before the species *C. elegans* was deliberately chosen by Sidney Brenner in the 1960s as a novel model organism to bridge the gap between the relatively simple unicellular organisms, like bacteria and phages, which were the main focus of molecular biology at the time, and more complex animals that were used in genetic studies, like *Drosophila* or mouse. “Thus we want a multicellular organism which has a short life cycle, can be easily cultivated, and is small enough to be handled in large numbers, like a micro-organism. It should have relatively few cells, so that exhaustive studies of lineage and patterns can be made, and should be amenable to genetic analysis” (proposal to the Medical Research Council, October 1963, as cited in ref. 46). In the end, he chose *C. elegans* for several reasons: The worms are 1 mm long and easy to culture in the laboratory on a diet of nonpathogenic *E. coli* bacteria; the life cycle only takes three days to four days at room temperature, and one adult worm can produce hundreds of offspring. Most *C. elegans* worms are self-fertile hermaphrodites, and only occasionally are males produced. Hermaphroditism greatly facilitates the generation of inbred lines and simplifies genetic screens, while the facultative males still allow specific crosses, for example, for mapping of mutations or the transfer of marker genes. Cultures of *C. elegans* can be frozen for prolonged storage; upon starvation, they enter into a quiescent stage, the “dauer” larva.

The first results from a genetic screen in *C. elegans* were published in 1974 (47). Several hundred ethyl methanesulfonate (EMS) induced mutants affecting behavior and morphology were described defining about 100 genes. The phenotypes of the mutants were mostly uncoordinated, roller, blistered, and dumpy or small. The mutants defined six linkage groups, and a genetic map was constructed. In parallel to genetic screens, the complete cell lineage of *C. elegans* was traced by direct observation under the microscope (48, 49). This established the invariance of the embryonic cell lineage in these animals. *C. elegans* worms have a fixed number of only 558 larval and 959 adult somatic cells which form the different organs, like cuticle-producing skin, muscles, or a simple nervous system. A large part of the body is occupied by the germ line–derived ovary in which, at the distal end, germ line stem cells produce first sperm and later oocytes which mature, get fertilized, and develop to an early cleavage stage before they are delivered via the vulva, a hermaphrodite-specific organ that develops during larval stages from four precursor cells. Although the invariance of cell divisions suggested a determination of cell fate via asymmetric cell divisions, in many instances, ablation of individual cells with a laser microbeam indicated inductive interactions between neighboring cells (50). In particular, the distal tip cell sitting at the end of the ovary was found to provide a stem cell niche: Ablation stopped the entire germ line from developing. This induction was found to depend on Notch signaling (reviewed in ref. 51). Many screens in the worm have been performed to unravel these cell-to-cell interactions with ever more sophisticated strategies and phenotypic readouts (reviewed in ref. 52). These have included modifier screens, enhancers or suppressors of an already present phenotype, and the use of green fluorescent protein (GFP)-marked strains to facilitate detection of subtle phenotypes. *C. elegans* was the first animal in which GFP was used as a marker for gene expression (53). The high numbers of individuals that can be assessed in *C. elegans* screens means that it is possible to reach saturation and to identify multiple mutant alleles of the same gene in one experiment. Therefore, hypomorphic (partial loss-of-function) and neomorphic (gain-of-function) alleles were recovered in many instances, making it possible to define the complete RAS pathway with mutations that lead to “vulvaless” or “multivulva” phenotypes (54). The possibility of obtaining temperature-sensitive alleles, and then using them in further screens, also proved to be of great value for *C. elegans* genetics. The first genes that regulate life span in a multicellular animal were identified in *C. elegans* in the background of temperature-sensitive alleles causing sterility or as temperature-sensitive alleles involved in dauer formation (55). Together, in many genetic screens, mutant alleles for several thousand genes were accumulated by the *C. elegans* community.

The invariance of the embryonic cell lineage in these animals revealed that a fixed number of cells die during development (131 in hermaphrodites) by programmed cell death or apoptosis. Many genes that are essential for the execution of the apoptotic program were first identified in *C. elegans* in genetic screens that made use of the advantages of this model organism (56).

In *C. elegans*, the first mutations in genes encoding small noncoding RNAs have been isolated—*lin-4* and *let-7*. These microRNAs regulate the translation of other genes by binding to regulatory elements in the target mRNA. These findings led to the discovery of RNAi: Injection of double-stranded RNA (dsRNA) molecules leads to specific degradation of a homologous mRNA and thereby to a knock-down of the gene function (57). A robust gene knock-down can be achieved for any gene—in the case of *C. elegans*, by soaking the worms in dsRNA or by feeding them bacteria that produce dsRNA (58).

*C. elegans* was the first animal to have its complete genome sequenced (59). The systematic knock-down of every known gene in genome-wide RNAi screens was now possible (60) and allowed the identification of ∼2,400 previously uncharacterized genes that give rise to mutant phenotypes. The core proteome of *C. elegans* consists of over 9,400 proteins (39), over half (56%) of which show homology to protein families also found outside nematodes (61). The genome encodes Hox genes and most of the signaling proteins known from *Drosophila*, with the notable exception of Hedgehog. (For a recent review on *C. elegans* developmental genetics, see ref. 44.)

## Danio rerio.

The zebrafish (*Danio rerio*) was first adopted as a model organism to study the development of a vertebrate via genetics by George Streisinger in Oregon (62). Having worked with bacteriophages in the 1950s and 1960s, Streisinger, similar to Sidney Brenner with *C. elegans*, wanted to establish a model system to study the development of the nervous system, however, not in one of the simplest animals but in a vertebrate. He chose the zebrafish (probably) for some of the same reasons other models had been chosen before, namely, the ease with which they can be bred in the laboratory, the large number of offspring that can be obtained from one individual, and the relatively short generation time of three months. In addition, the early embryos of zebrafish are almost completely transparent, which makes it possible to follow development directly under a microscope. In the beginning, methods for the generation of homozygous zebrafish lines were established involving heat shock or high pressure applied to cleavage-stage haploid embryos; these methods allowed screening for recessive mutations in haploids or diploid gynogenetic offspring (i.e., fish derived solely from a female), thus considerably speeding up the process of isolation of recessive mutants (63). Although attractive on paper, the method has not proven practical because of a high background of unspecific lethal embryos. The first documented mutations in zebrafish (*golden*, *brass*, and *albino*), which could be used as genetic markers, arose spontaneously in zoo and laboratory populations. These mutations affect the pigmentation of black melanin–containing cells, the melanophores, easily visible in larvae just a few days old. After the untimely death of George Streisinger in 1984, groups in Oregon carried on with zebrafish research, and, a few years later, other groups in Europe and the United States (Christiane Nüsslein-Volhard in Tübingen, and Marc Fishmann, joined by Wolfgang Driever, in Boston) began to see the potential in the fish for genetic analysis of development (62). The first zygotic lethal mutation in zebrafish (*spadetail*) was described and published in 1989 (64), and it seemed possible to address development of a vertebrate with genetic methods, similar to the great success in *Drosophila* (62). There was a fear, however, that the mutability might not be high, because teleost fish had undergone an extra genome duplication, and therefore the genome was expected to contain many duplicated genes, mutations in which would be compensated by a paralog. Methods for large-scale mutagenesis were developed for zebrafish, and it was found that ENU (ethylnitrosourea) was a mutagen that induced mutations at about the same rate as EMS in *Drosophila* (65, 66).

Two large-scale screens for mutations that affect a broad variety of developmental processes were carried out as communal efforts simultaneously in Tübingen and Boston in the early 1990s (67, 68). Families were raised from crosses between two individuals heterozygous for a mutagenized genome, and eggs from several F2 crosses were collected and inspected for mutant phenotypes until day 5, when the fish larvae develop a swim bladder and begin to feed. As the zebrafish embryo does not shed a cuticle but is composed entirely of soft tissue that decays quickly after death, it was necessary to inspect the clutches almost every day to not miss early lethal phenotypes. On the other hand, the transparency allowed a large number of organs and tissues to be scored simultaneously. Together, these screens yielded about 1,500 mutations in approximately 400 genes published in 37 papers in one issue of *Development* in 1996 (69), which laid the foundation for much of the research carried out on the development of zebrafish in the years that followed. Of great advantage for all these studies of development was the fact that zebrafish develop very rapidly, and the larvae are transparent, even beyond two days of development in *albino* or *nacre* mutants, which lack pigmented melanophores. Already 12 h after fertilization, the larval body plan is established, and, after 24 h, many organs (brain, muscles, and notochord) can be identified. Zebrafish can be used as a model to understand vertebrate development through genetics, as the development of many organs and tissues such as heart, vasculature, blood, kidney, eyes, inner ear, somites, notochord, jaw and branchial arches, liver, and brain can be clearly followed in the living embryo.

With the establishment of the Tol2 system as an efficient method to generate transgenic zebrafish (70) and the introduction of GFP-labeled cells, the transparency of the zebrafish larvae proved, once more, tremendously advantageous, allowing the very detailed analysis of mutant embryos with fluorescent marker lines. The Gal4/UAS system of targeted expression was adopted from *Drosophila*, and the Cre/loxP system was adopted from mice. Zebrafish has also proven useful in analyzing nerve connections directly, or by using touch response, vision, or hearing assays; many sophisticated behavioral studies are now carried out in zebrafish larvae (71). Zebrafish are also used to study the etiology of many human diseases in great detail (72). There are models for a large variety of developmental disorders and cancer (73).

Zebrafish are used as a model to study pigment pattern formation. The striped pattern of the adult fish, which is produced by three types of neural crest–derived pigment cells, develops during metamorphosis. A number of patterning genes have been identified by defects in stripe formation, some of which are involved in direct cell contacts among the pigment cells (74). Closely related *Danio* species, which often display very different patterns, offer the unique opportunity to identify evolved genes and to reconstruct the evolutionary history of biodiversity in vertebrates (75, 76).

Besides zebrafish, another frequently used fish species for laboratory research is medaka (*Oryzias latipes*), the Japanese rice fish. Despite superficial similarities between zebrafish and medaka, they are only distantly related and, therefore, can be viewed as models that complement each other quite well.

## Mus musculus.

Very soon after the rediscovery of Mendel around 1900 by plant breeders, Mendelian inheritance was demonstrated for the *albino* locus in the house mouse (77). This was possible because “mouse fanciers” had kept and bred several lines with clearly distinguishable traits; most prominently among these characteristics were different coat colors. A dominant yellow mutation with a homozygous lethal phenotype was found, and, later, linkage of two genes, *pink-eye* and *albino*, was demonstrated, thus allowing more-confident generalizations about genes and chromosomes from *Drosophila* to other organisms. The realization that the genetic background might influence the phenotype ultimately led to the generation of laboratory strains from the available fancy lines through multiple generations of inbreeding, the first one being DBA (dilute, brown, nonagouti). Today, all inbred strains used in the laboratory are derived from a limited subset of haplotypes from three different subspecies, *M.m. domesticus*, *M.m. musculus*, and *M.m. castaneus* (78). From early on, cancer susceptibility of mice and a possible genetic influence was recognized, and remained one of the main research topics for several decades. The realization that mammary tumors, which occurred spontaneously at high frequencies in some strains, were transmitted through weaning eventually led to the discovery of the mouse mammary tumor virus, viral insertions in the genome, and, ultimately, cellular proto-oncogenes. Tumor transplantation experiments in mice led to the discovery and analysis of the major histocompatibility complex (reviewed in ref. 79).

Being mammals, mice can be closer models for human genetics, development, and disease than flies or worms. This turned out to be the case for sex determination and dosage compensation. Sex in *Drosophila* or *C. elegans* is determined by the ratio of X chromosomes to autosomes; worms do not have a Y chromosome, and, in flies, the Y chromosome only contains genes required for male fertility. In contrast, it was found that, in the mouse, the presence of a Y chromosome determines male development of the embryo. The dominant factor on the Y chromosome, the “testis-determining Y gene” (*Tdy*), was found to be equivalent to *Sry* (*Sex-determining region Y*) (80). The *Sry* gene encodes a transcription factor that acts in the developing gonad to induce testis development; subsequently, hormones produced by the testis determine male differentiation of the embryo. This system is conserved in all eutherian mammals, including humans (81). The X chromosome dosage compensation, that is, the process used to compensate for the fact that males have only one X chromosome whereas females, or hermaphrodites in *C. elegans*, have two, is also markedly different between flies, worms, and mammals. In hermaphroditic worms, transcription rates from both X chromosomes are reduced; in flies, they are increased from the single X chromosome present in males. In mammals, a different mechanism is used. Already in 1961, Mary Lyon (82) suggested that, in female mice, during early embryonic development, one of the two X chromosomes is randomly picked and inactivated, a hypothesis later shown to be true for all eutherian mammals (83).

During the first decades of the 20th century, a growing number of mutants spontaneously arising in mouse colonies were collected, many of them affecting coat color or other obvious traits. Some examples are dominant-white spotting (*W*) and Steel (*Sl*), which code for the receptor and protooncogene c-kit (84) and its ligand (85), respectively. Further examples are *obese* (*ob*) and diabetic (*db*) mutants; homozygous *ob* or *db* mutants are characterized by excessive feeding, weight gain, and morbid obesity. The *ob* gene codes for the hormone leptin that signals satiety (86); *db* codes for the corresponding receptor (87). Another mutation that arose spontaneously, *brachyury* (*T*), affects tail length and sacral vertebrae in heterozygous animals, leading to embryonic lethality in homozygotes. The *T* gene encodes a highly conserved transcription factor important for mesoderm development (88). Systematic unbiased mutant screens can only be carried out at a slow pace in mice (compared with worms and flies), owing to the small litter sizes, the difficulty of analyzing the mutant embryos in utero, and the considerable space requirements for large mouse colonies.

Mouse genetics changed dramatically when ES cells were developed (89, 90), with the possibility of inducing specific mutations by homologous recombination, and a field of reverse genetics was created. Embryonic development in mouse, like in all mammals, occurs in two phases: The mouse egg is tiny, it is fertilized by sperm in the oviduct, and then it develops into a blastocyst composed of an inner cell mass (ICM) of about 100 undifferentiated cells surrounded by a syncytial epithelium, the trophectoderm. The blastocyst, upon implantation, attaches to the uterus, and the ICM will develop into the embryo and extraembryonic membranes, whereas the trophectoderm contributes to the placenta. In special culture media, ICM cells can be propagated while retaining their undifferentiated pluripotent state; upon transplantation into an early mouse embryo, they can contribute to all tissues of the host including the germ line. In these cultured ES cells, plasmids carrying mutations in a cloned gene can be introduced, and, by (rare) homologous recombination events, the mutant copy can integrate into the genome. Positive and negative selection systems were developed to identify the cells with the desired recombination events (91). Introduction of the mutated ES cells into a blastocyst then can generate mosaic mice which may propagate the mutant gene copy to their progeny. Homozygous mutant embryos will occur after inbreeding in the next generation.

The creation of knock-out mice by “reverse genetics” allowed testing of the contribution of genes known from other organisms such as *Drosophila* or *C. elegans* in the mouse. To overcome developmental lethality, tissue-specific knock-outs using the Cre/loxP system were produced. The Cre recombinase gene from a bacteriophage under the control of a tissue-specific promoter was introduced (92). Combining the recombinase in one individual with a gene flanked by loxP sites (floxed) leads to the removal of the floxed DNA sequence, resulting in a mutant allele (93). The system was further refined by the introduction of a ligand-inducible Cre recombinase (CreER^T^) to achieve temporal control (94). In addition to the Cre/loxP system, a method to reversibly switch promoters on or off by providing exogenous tetracycline was established (95). For a long time, the mouse was the only system in which the technology of reverse genetics via ES cells could be achieved, and attempts to create ES cells for other mammals, for example, rats, remained largely unsuccessful for many years (96). Today, human ES cells can also be generated (97), and induced pluripotent cell lines from somatic cells can be used to create genetically modified organ cultures (98). With the introduction of the CRISPR-Cas system, it became much faster and easier to generate knock-out mice by directly injecting the guide RNA and Cas9 enzyme into embryos, and, also, repair of mutations is possible with high efficiency.

## Arabidopsis thaliana.

Like *Drosophila*, the small weed *Arabidopsis thaliana* has no commercial value and is usually regarded as more of a nuisance than anything positive. Nevertheless, it became the leading plant for scientific research in the decades since the 1980s. Research with *A. thaliana* began, very similar to *Drosophila*, in the early years of the 20th century with the question of how chromosomes behave in the cell nucleus and how they relate to heredity (99). However, unlike *Drosophila*, in the following decades, very few scientists chose to work with *Arabidopsis*, and very little was published. Instead, maize (corn) developed into a genetic model organism. In 1901, Correns (100), one of the rediscoverers of Mendel’s work, published his results from crosses of maize varieties with differently colored endosperm. Later, the cytological demonstration of crossover (101), and the identification of transposable elements (102) followed. Genetic screens in maize were used to uncover genes for important agronomic traits, and it became an important model for quantitative genetics and to understand heterosis (hybrid vigor) (103). However, with the beginning of molecular biology, when the first genes were cloned, the small genome size of *Arabidopsis* (with approximately 135 Mbp, similar to that of *C. elegans*, 100 Mbp, or *Drosophila*, 180 Mbp) became a very distinct advantage over most other plants (which are often polyploid), for example, compared to maize, 2.4 Gbp, or barley, 5.3 Gbp. In 1975, Rédei (104) suggested using *Arabidopsis* as a genetic tool, stating that “the major advantage of *Arabidopsis* is that it can be subjected to manipulations common to microorganisms, which are impractical in most other higher plants.” In addition to being small, fast, and self-fertilizing, the possibility of saturation mutagenesis was recognized as another clear advantage. It now seemed possible to study all kinds of processes in great detail through the analysis of mutants (105).

*Arabidopsis* is a self-fertilizing dicotyledonous flowering plant. Unlike animals, plant cells are surrounded by a rigid cell wall, and growth takes place in special regions of the plant, the meristems, in which the cells remain multipotent and retain the ability to divide and sprout. Cells from the meristem can give rise to a new plant, so vegetative propagation is common in plant breeding. Plants have a remarkable power of regeneration, as whole plants can be derived from undifferentiated callus cultures. The flowers are composed of four whorls of flower organs—the sepals (at the outer base), petals (making the flower), stamen (making pollen), and carpels (containing eggs). The identity of these flowering organs is determined by a combinatorial set of MADS-box transcription factors which act similarly to the homeotic genes in animals; the ground state is leaf (106). Fertilization occurs in the carpels by two pollen nuclei, one of which fuses with the egg cell to give rise to the zygote; the other fuses with an associated cell to produce the triploid endosperm, which serves as nutrition for the embryo. The cells in the embryo divide to form a seedling which, essentially, is composed of two primary leaves, the cotyledons, an apical (shoot) and basal (root) meristem. The seed contains the embryo in a dormant state; it will germinate in the next season, but can be stored for long times.

Mutagenesis is achieved by soaking the seeds in an EMS solution. Saturation mutagenesis screens have been carried out for processes of basic cell biology of plants, nutrition requirements, flower development, root development, floral induction, circadian rhythm, embryogenesis and seed formation, light perception, cold and heat resistance, hormone action and others (107). A screen for embryo-patterning mutants identified nine genes involved in either apical–basal organization into shoot meristem, hypocotyl, and root meristem; the radial organization of epidermis; ground tissue and vascular tissue at the center; or the shape of the embryo (108). Plants have a sophisticated genetic system of defense against infection, similar to the innate immune system of animals. Resistance genes operate in a similar manner in all plants, so research on resistance in *Arabidopsis* is applicable to convey resistance to crops (109).

The first transgenic plants were tobacco (110); however, very soon, the *Agrobacterium tumefaciens*–based method was applied to *Arabidopsis* (111), and, crucially, it turned out that a very much simplified “floral dip” method could be used to obtain stable transgenic lines in *Arabidopsis*, thus eliminating tissue culture and plant regeneration (112). This method was used to create a collection of more than 200,000 mutant lines carrying transfer DNA insertions in almost every single gene in *Arabidopsis*. In 2000, the complete genome sequence of *Arabidopsis* was published (113). Soon afterward, the 1001 Genomes Project was launched, aiming to obtain complete genome sequences for 1,000 additional *Arabidopsis* strains (“accessions”) in an effort to sample the natural variation present in diverse geographical locations (114). These natural strains of *Arabidopsis* are basically inbred lines, which makes it possible to use them in genome-wide association studies to investigate the genetic basis of different traits (115), many of which are important for agriculture in a variety of different plant species.

## Discussion

When, in the 1970s, the systematic mutant screens in the two small invertebrate models *Drosophila* and *C. elegans* began, and communities of geneticists started to form around them, the idea was to use mutations as an unbiased approach to identify genes encoding key determinants and to unravel regulatory pathways that govern development. A mutation allows the elimination of a single component in a complex system while leaving everything else intact. Genetic interference avoids the disturbances usually following experimental interference, which had been so frustrating in other developmental systems, for example, in the attempts to isolate the physical basis of the organizer. However, the aspect of serving as models for “higher” organisms with relevance even for human health, which is so prevalent in present-day biology, was far from anyone’s thoughts. It seemed highly unlikely that similarities existed between the genetic regulation of vertebrates and invertebrates, because they displayed vastly different morphologies and developmental strategies.

The choice of the zebrafish for systematic genetic analysis rested on the conviction of the power of genetics to dissect complex processes also in a vertebrate and the ability to compare organisms on the basis of their genes. The discovery of the grand homologies which followed the molecular cloning of many developmental genes from *Drosophila* and *C. elegans* initially came as a great surprise but, later, was confirmed by many zebrafish mutants that supported the homology of fly, frog, and mouse development. Analysis of mammalian development profited much from reverse genetics, starting with cloned genes of the small invertebrate models, and visualizing important processes in the transparent zebrafish can also help in understanding mammalian development. It is important to note that the discovery of several developmental principles of general importance rested on some special features of the small invertebrate models: Syncytial development in *Drosophila* led to the discovery of morphogenetic gradients and transcription factor hierarchies, and the fixed small cell number and short life cycle in *C. elegans* led to the discovery of programmed cell death, RNAi, and control of longevity. Research on the plant model *Arabidopsis* has resulted in the development of technologies that allow genetically modification of crop plants. With these tools available, it is likely that a number of other organisms will develop into “models” with genetic analysis of specific aspects by a growing community of researchers.

After the first whole genome sequences (WGS) of multicellular animals were available for *C. elegans*, *Drosophila*, and humans, it came as a surprise that the numbers of genes in all three species are quite similar, despite the 30-fold difference in genome size. Whereas humans have approximately 24,000 protein-coding genes, the numbers for *C. elegans*, approximately 20,000, and *Drosophila*, approximately 14,000, are not much lower. The increase in organismal complexity therefore did not occur simply by an increase in the number of genes. But size and complexity of genes increased, with more splice isoforms or other variations of posttranscriptional and posttranslational modifications, and a higher complexity in the domain composition of proteins. The general conclusions, well confirmed in the WGS projects, are, first, that there are by far fewer genes than features/traits which can be affected by a mutation, which means that every cell/tissue/process depends on very many genes, and, second, that most genes are involved and active in different forms and modifications in many different contexts. All models that have been extensively and productively used in forward genetic screens, *C. elegans*, *Drosophila*, *D. rerio*, and *Arabidopsis*, have in common that they show low levels of genetic redundancies, that is, genes with (almost) identical functions where one could compensate for the loss of the other. *Drosophila* with only about 5,000 genes with paralogs, seems to have the lowest level of genetic redundancy, making forward genetic screens especially efficient.

The zebrafish and mouse genomes have a gene content very similar to humans, with 26,000 and 23,000 protein-coding genes, respectively. Especially for zebrafish, this means that some of the early apprehensions that the teleost-specific whole genome duplication might have produced many paralogous genes with still very similar functions were finally refuted. In fact, loss or subfunctionalization of two paralogs derived from one common ancestral gene is frequent, and it can lead to a better understanding of gene function, as the phenotypes of the mutants might be easier to interpret under these less pleiotropic circumstances. The functions of many of the genes identified in the simpler systems like *C. elegans* or *Drosophila* were studied by knock-out in the mouse. The surprising finding that some genes that were deemed “important” did not result in significant knock-out phenotypes might indicate that these were too subtle to be detected under laboratory conditions, or that the contribution to a particular trait was compensated by paralogs or the activity of other genes. Genes mutating to a clean and distinct phenotype are the exception rather than the rule. Sometimes, identical mutations were found to result in rather different phenotypes, depending on the mouse strain, that is, the genetic background, highlighting the polygenic nature of gene functions.

Inbred strains that are nearly isogenic exist for the mouse, due to the early selection in cancer susceptibility and transplantation experiments. Most *C. elegans* and *Arabidopsis* isolates are highly inbred because these species are self-fertilizing hermaphrodites. In *Drosophila*, highly inbred strains have been available since the construction of balancer chromosomes; before mutagenesis, the relevant chromosome was usually isogenized to ensure that no background mutations were present. In zebrafish, however, rigorous inbreeding generally leads to reduced fecundity and lower vigor. Zebrafish strains are usually bred in larger populations to maintain some heterozygosity but are tested for the absence of background mutations before mutagenesis.

Genomic analysis opened new avenues for developmental genetics in allowing quantification of expression by RNA sequencing and proteomics, and even single-cell transcriptomics. Although it does not necessarily provide means to assess the significance of the contribution of particular genes, it will certainly complement the unbiased genetic approaches. The focus on genetic in-depth analysis in a few selected organisms will remain substantial for advances in many fields of biology.

## Data Availability

There are no data underlying this work.
